# Immunosuppression and transplantation-related characteristics affect the difference between eGFR equations based on creatinine compared to cystatin C in kidney transplant recipients

**DOI:** 10.1093/ckj/sfae253

**Published:** 2024-08-20

**Authors:** Lukas Weidmann, Catherine Laux, Kai Castrezana Lopez, Dusan Harmacek, Britta George, Seraina von Moos, Thomas Schachtner

**Affiliations:** Department of Nephrology, University Hospital of Zurich, Zurich, Switzerland; Department of Nephrology, University Hospital of Zurich, Zurich, Switzerland; Department of Nephrology, University Hospital of Zurich, Zurich, Switzerland; Department of Nephrology, University Hospital of Zurich, Zurich, Switzerland; Department of Nephrology, University Hospital of Zurich, Zurich, Switzerland; Department of Nephrology, University Hospital of Zurich, Zurich, Switzerland; Department of Nephrology, Cantonal Hospital of Lucerne, Lucerne, Switzerland; Department of Nephrology, University Hospital of Zurich, Zurich, Switzerland

**Keywords:** creatinine, cystatin C, estimated glomerular filtration rate, immunosuppression, kidney transplantation

## Abstract

**Introduction:**

Previous studies show heterogeneity when applying estimated glomerular filtration (eGFR) equations to kidney transplant recipients (KTRs). However, research on the impact of transplantation-related characteristics on eGFR equations using creatinine (eGFRcr) compared to cystatin C (eGFRcys) is scarce.

**Methods:**

We conducted a comprehensive analysis with three eGFRcr equations (Chronic Kidney Disease Epidemiology Collaboration (CKD-EPI) 2009, European Kidney Function Consortium (EKFC) 2021, kidney recipient specific-glomerular filtration rate KRS-GFR) 2023), comparing them to two eGFRcys (CKD-EPI 2012 and EKFC 2023) in 596 KTRs. Bland–Altman plots demonstrated relative differences according to different eGFR-stages. Multivariable logistic regression identified transplantation-related characteristics independently associated with smaller or greater differences between eGFRcr and eGFRcys equations.

**Results:**

94.3% of the cohort were White individuals. Median eGFR differed as much as 9 ml/min/1.73 m^2^ between equations. The median relative differences (Q2) were greater (more negative) when comparing the eGFRcr equations to eGFRcys CKD-EPI 2012, than when comparing them to eGFRcys EKFC 2023 (*P* < .001). Better average eGFR was associated with smaller mean relative differences in all comparisons but eGFRcr CKD-EPI 2009 with eGFR EKFC 2023 and eGFRcr EKFC 2021 with eGFRcys EKFC 2023. Living kidney donation and belatacept use were independent factors associated with a smaller difference (≥Q3) between eGFRcr and eGFRcys equations, while prednisone use or higher HbA1c were independently associated with a greater difference (≤Q1) between equations.

**Conclusion:**

Different eGFR-stages, donor, or recipient characteristics, along with immunosuppression such as belatacept or prednisone, contribute to differences between eGFRcr and eGFRcys. These effects need to be considered in the clinical management of KTRs.

## Graphical Abstract

**Graphical Abstract:**
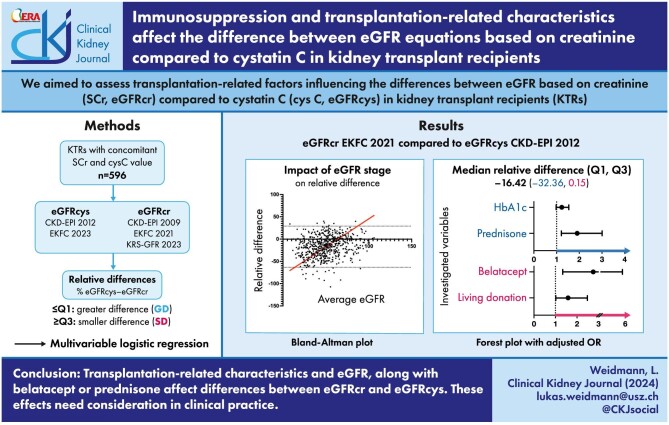

KEY LEARNING POINTS **What was known:**

- Equations used to estimate glomerular filtration rate based on creatinine or cystatin C have primarily been validated in individuals who have not undergone kidney transplantation.
- The performances and accuracies of estimated glomerular filtration rate equations exhibit heterogeneity when applied to kidney transplant recipients and compared to measured glomerular filtration rate.
- Both creatinine and cystatin C are influenced by different factors specific for the setting of kidney transplantation, such as body composition of the donor or recipient, and other transplantation-specific characteristics.

**This study adds:**

- A comparative analysis of the most pivotal equations to estimate glomerular filtration rate based on creatinine compared to estimated glomerular filtration rate equations using cystatin C.
- Examining transplantation-related characteristics, including donor and recipient attributes, as well as immunosuppression, to better understand their influence on the disparities observed between the aforementioned equations.

**Potential impact:**

- Information on factors influencing the differences of equations to estimate glomerular filtration rate based on creatinine compared to equations based on cystatin C in the population of kidney transplant recipients.
- The study raises awareness of potential circumstances under which a greater divergence or agreement between the formulas can be expected, which potentially impacts the clinical management of kidney transplant recipients.

## INTRODUCTION

Since the 1970s, assessing kidney function through creatinine-based estimation of glomerular filtration rate (GFR) has become

a routine in nephrology [1, 2]. A precise estimation of GFR (eGFR) holds significance for drug dosing and effective management of patients with chronic kidney disease (CKD), including kidney transplant recipients (KTRs) [3, 4]. Even though there is a variety of equations to calculate eGFR based on creatinine (eGFRcr), most of them have been created in non-transplanted patient cohorts and validated based on measured GFR (mGFR) [5–7, 8]. Moreover, the recommendations on the choice of the preferred eGFR equation in nephrology changes depending on different geopolitical perspectives, creating discrepancies between European and American practices. For instance, in the USA, the preference leans towards the ‘Chronic Kidney Disease Epidemiology Collaboration’ (CKD-EPI) 2021 equation rather than CKD-EPI 2009, which remains the current standard in Europe, regardless of the race coefficient [9], [10]. However, the ‘European Kidney Function Consortium’ (EKFC) has developed its own two distinct formulas: one using creatinine (EKFC 2021) and another employing cystatin C, or a combination of both (EKFC 2023) [8], [11]. The latest KDIGO guidelines (April 2024) recommend using the most suitable ‘regional choice’ as the standard for eGFR. In Europe, this could be either the CKD-EPI 2009 or the EKFC 2021 formula [12]. Previous studies investigating the application of eGFR equations in KTRs have shown heterogeneity in their performance compared to measured GFR (mGFR) [13–15, 16]. Therefore, a recently published, race-free, ‘kidney recipient specific’ eGFRcr (KRS-GFR) equation especially for KTRs was designed in hope to surpass irregularities [17]. It performed similarly to current eGFRcr equations (e.g. CKD-EPI 2009 or 2021), however showing variance in performance when tested in different validation cohorts. Additionally, the validation of the new equation lacked the consideration of cystatin C, which is a suggested additional parameter in situations of uncertainty due to non-GFR-determinants of creatinine [18], [19]. Differences between patients with native and transplanted kidneys significantly impact the accuracy of eGFR equations because of various factors such as donor-recipient mismatch, immunosuppressive treatments (such as corticosteroid use), and the presence of a single kidney metabolism [20], [21]. These factors influence creatinine and cystatin C. The differences of eGFRcr equations compared to eGFRcys equations in respect to the aforementioned influencing parameters have not been well described, especially considering newer eGFR equations in KTRs. A recent study conducted with CKD patients demonstrated a relevant influence of disparities between eGFRcr and eGFRcys on outcomes, including death, highlighting the importance of these considerations [22]. In our investigation, utilizing a well-defined cohort of ∼600 KTR transplanted at the University Hospital of Zurich between 2009 and 2021, we analysed influencing factors for the difference of three eGFRcr equations (CKD-EPI 2009, EKFC 2021, KRS-GFR 2023) compared to two eGFRcys (CKD-EPI 2012, EKFC 2023). Our assessment encompassed a comprehensive spectrum of transplantation-related variables, including recipient characteristics, donor characteristics, and immunosuppression.

## MATERIALS AND METHODS

### Study cohort

**Figure 1:**
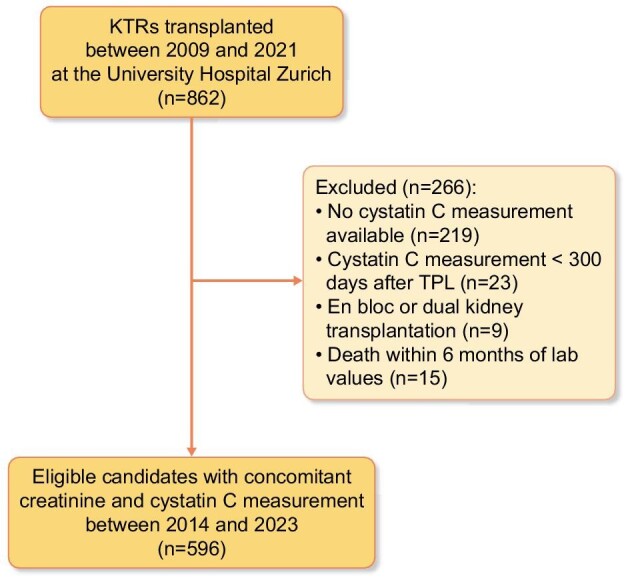
Study flowchart demonstrating the deduction of the study cohort. Exclusion criteria are listed. Abbreviation: TPL, transplantation.

Out of 862 candidates transplanted between 2009 and 2021 at our center (University Hospital of Zurich), 596 KTRs were extracted for this non-prespecified analysis. All patients provided general consent for the use of their clinical data. The study was conducted in accordance with the Declaration of Helsinki and approved by the cantonal ethics committee (BASEC 2019–01274). Relevant demographic and biometric variables were available for both recipients and donors. Inclusion criteria were age >18 years and an accessible contemporaneous creatinine and cystatin C measurement at least 300 days after transplantation (in steady state). Exclusion criteria were creatinine and contemporaneous cystatin C measurement only <300 days after transplantation, the presence of acute kidney injury as compared with two other values of creatinine within 6 months, death within 6 months of measurement, en bloc- or double-kidney transplantations (more than one functioning kidney), and missing cystatin C measurements. The deduction of the study cohort is presented in Fig. 1.

### Patient follow-up

After transplantation, all KTRs underwent follow-up according to the subsequent schedule: weekly from week 2–6, biweekly from week 6–12, monthly from month 3–6, and every 2 months from month 6–12. Afterwards, ongoing care was provided every 3 months in collaboration with local nephrologists and at least annually at our outpatient clinic.

### Assessed eGFR equations

The equations assessed were the CKD-EPI 2009 equation, the EKFC 2021 equation, and the recently published KRS-GFR 2023 equation [6, 8, 17]. The analyzed eGFRcys equations used as references for the analysis were the CKD-EPI 2012 and EKFC 2023 equations [7, 11, 23]. De-indexing for body surface area (BSA) was applied to demonstrate median eGFR with the ‘du bois and du bois method’ (Appendix 1) [24].

### Assessment of difference between the equations

Difference was calculated by subtracting the investigated eGFRcr equation from the reference eGFRcys equation. Consequently, a negative difference signifies that eGFRcr overestimates the GFR compared to eGFRcys (reference), while a positive difference suggests that the eGFRcr underestimates the GFR compared to the reference. Bland–Altman plots were used to demonstrate the relative differences (including 95% limits of agreement) according to different eGFR-stages (average eGFR of the compared equations). Deming regression with standardization was performed to demonstrate the mean relative difference for every eGFR-stage as a linear function. For the binary logistic regression analysis investigating the influencing variables on eGFR disparities, difference was also assessed using the relative difference of the eGFR equations (‘eGFRcys minus eGFRcr divided by their average and multiplied with 100%’). As all median (=Q2) relative differences comparing eGFRcr equations to eGFRcys were negative, all values ≤Q1 were associated with a greater difference between eGFRcr and the reference, while values ≥Q3 were associated with a smaller difference (or more beneficial estimation of the eGFRcys equations regarding kidney function).

### Laboratory assessment of creatinine and cystatin C

Serum creatinine and cystatin C values from July 2014 until March 2023 were included for the analysis. Serum creatinine measurements were performed with the modified Jaffe reaction (including ‘rate-blanking’ to reduce disruptive factors) on a Roche Cobas 8000 (c702 Module) using the manufacturer's reagents. Coefficients of variation were 2.4% at 1.1 mg/dl (94 µmol/l) and 2.3% at 4.0 mg/dl (357 µmol/l). Serum cystatin C measurements were measured with Roche Cobas 8000 (c502 Module) with a latex-enhanced immunoturbidimetric assay. Coefficients of variation were 1.3% at 1 mg/l and 1% at 4.01 mg/l. All measurements of serum creatinine or cystatin C investigated in this study have been traceable to the IDMS standard or to IFCC reference materials, respectively.

### Statistical analysis

Statistical analysis was performed using IBM SPSS Version 29 (SPSS, Chicago, IL, USA), Microsoft^®^ Excel^®^ 2016 (^©^2010 Microsoft Corporation. All rights reserved) and GraphPad Prism Version 9.5.1. Comparison of medians included the application of the Mann–Whitney *U*-test (for two variables) of the Kruskal–Wallis test (more than two variables). Odds ratios (OR) with 95% confidence intervals (CI, low and high) were calculated using binary logistic regression models assessing transplantation-related characteristics. Multivariable analysis included antecedent univariable testing of all relevant variables included in the analysis. Only variables reaching a *P* value of <.05 were included into multivariable regression. To minimize multicollinearity, a correlation matrix using a Spearman’s rho test was performed (Appendix 2). Variables with a correlation coefficient (*r* value) of ≥.7 have not been tested in the same model. Continuous variables were described using median and IQR (Q1–Q3) regardless of distribution. Categorical variables were presented as percentages of total (%). A *P* < .05 was considered significant.

## RESULTS

### Baseline and transplantation-related characteristics

Table 1 demonstrates the baseline characteristics and Table 2 the transplantation-related characteristics of the study population. Most KTRs, namely 562 (94.3%) individuals were White. Only 14 (2.3%) patients were Black, 14 (2.3%) were Asian, and six (1%) were Hispanic. Similar rates were observed in the donor group (97.1%, 0.3%, 2.3%, and 0.2%, respectively). The most common immunosuppressive regime consisted of tacrolimus, mycophenolate, and low dose prednisone (5 mg) (82.7%, 89.8%, and 61.4%, respectively). The median number of months after transplantation at which laboratory values were collected was 49 (24–97).

**Table 1:** Baseline characteristics of the study population: Continuous variables are presented as median (IQR), categorical variables as count (percentage).

	All (*n* = 596)
**Recipient**	
Sex	
Female *n (%)*	237 (39.8)
Male *n (%)*	359 (60.2)
Ethnicity	
Asian *n (%)*	14 (2.3)
Black *n (%)*	14 (2.3)
Caucasian *n (%)*	562 (94.3)
Hispanic *n (%)*	6 (1)
Age (years)	
At transplantation *median (IQR)*	52 (40–60)
At laboratory values *median (IQR)*	56 (45–65)
Biometrics	
Height (cm) at transplantation *median (IQR)*	170 (163–177)
Weight (kg) at transplantation *median (IQR)*	72 (61–84)
BMI (kg/m^2^) at transplantation *median (IQR)*	24.7 (21.7–27.9)
Weight (kg) at laboratory values *median (IQR)*	73 (62–85)
BMI (kg/m^2^) at laboratory values *median (IQR)*	24.9 (21.8–28.5)
BSA (m^2^) at laboratory values *median (IQR)*	1.84 (1.68–1.99)
Underlying disease type	
ADPKD *n (%)*	114 (19.1)
CAKUT *n (%)*	38 (6.8)
Hypertensive nephropathy *n (%)*	42 (7)
Diabetes type 1 *n (%)*	46 (7.7)
Diabetes type 2 *n (%)*	30 (5)
Glomerulonephritis *n (%)*	179 (30)
Others *n (%)*	71 (11.9)
Unknown *n (%)*	76 (12.8)
**Donor**	
Sex	
Female *n (%)*	280 (47)
Male *n (%)*	316 (53)
Ethnicity	
Asian *n (%)*	14 (2.3)
Black *n (%)*	2 (0.3)
Caucasian *n (%)*	579 (97.1)
Hispanic *n (%)*	1 (0.2)
Age (Years)	
At transplantation *median (IQR)*	53 (42–61)
Allograft age at laboratory values *median (IQR)*	57 (47–66)
Biometrics	
Height (cm) at transplantation *median (IQR)*	170 (165–180)
Weight (kg) at transplantation *median (IQR)*	75 (65–84)
BMI (kg/m^2^) at transplantation *median (IQR)*	24.9 (23–27.8)

Abbreviations: ADPKD, autosomal-dominant polycystic kidney disease; CAKUT, congenital anomalies of the kidney and urinary tract.

Others: Alport syndrome, hemolytic uremic syndrome, amyloidosis, chronic interstitial nephritis, tubulopathies, and end stage renal disease after shock.

**Table 2:** Transplantation-related characteristics of the study population. Categorical variables are shown as count (percentage).

	All (*n* = 596)
Transplantation type	
Living *n (%)*	172 (28.9)
Deceased *n (%)*	424 (71.1)
Transplantation subtype	
cABO *n (%)*	140 (23.5)
iABO *n (%)*	27 (4.5)
DBD *n (%)*	278 (46.6)
DCD *n (%)*	82 (13.8)
SPK *n (%)*	40 (6.7)
Others *n (%)*	29 (4.9)
Function after transplantation	
Delayed graft function *n (%)*	123 (20.6)
Number of transplantations	
First transplantation *n (%)*	513 (86.1)
Second transplantation *n (%)*	73 (12.2)
More than two transplantations *n (%)*	10 (1.7)
Immunosuppression	
Tacrolimus *n (%)*	493 (82.7)
Ciclosporin *n (%)*	45 (7.6)
Sirolimus/everolimus *n (%)*	30 (5)
Mycophenolate *n (%)*	535 (89.8)
Azathioprine *n (%)*	36 (6)
Prednisone *n (%)*	366 (61.4)
Belatacept *n (%)*	45 (7.6)
Donor-recipient-sex-mismatch	
D–R sex match *n (%)*	297 (49.8)
D–R sex mismatch *n (%)*	299 (50.2)
Donor–recipient sex ratio	
Female to female (FF) *n (%)*	109 (18.3)
Female to male (FM) *n (%)*	171 (28.7)
Male to male (MM) *n (%)*	188 (31.5)
Male to female (MF) *n (%)*	128 (21.5)
Donor–recipient BMI mismatch	
D-BMI > R-BMI (>20%) *n (%)*	89 (14.9)
D-BMI ∼ R-BMI (within 20%) *n (%)*	385 (64.6)
D-BMI < R-BMI (>20%) *n (%)*	114 (19.1)
Missing data (unknown) *n (%)*	8 (1.3)

Abbreviations: cABO, ABO-compatible kidney transplantation; iABO, ABO-incompatible kidney transplantation, DBD, donation after brain death; DCD, donation after circulatory death; SPK, simultaneous pancreas-kidney transplantation; mTOR, mammalian target of rapamycin.

Others: KaLuT (kidney after lung transplantation), KALT (kidney after liver transplantation), SIK (simultaneous islet and kidney transplantation), SLK (simultaneous liver and kidney transplantation)

### Median eGFR of all equations

**Figure 2:**
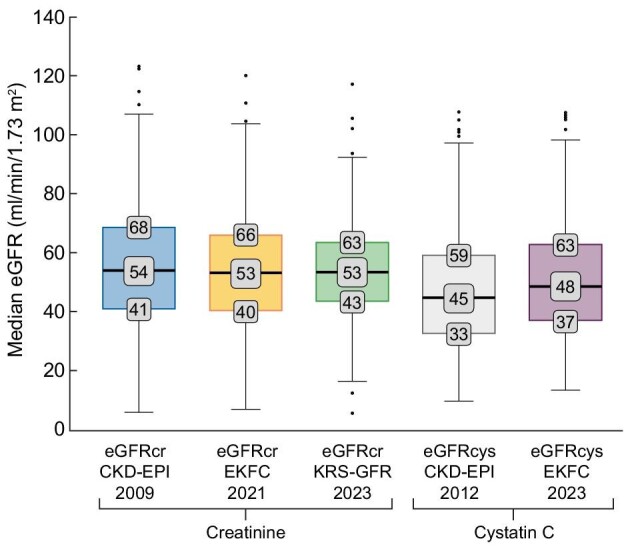
Median eGFR values according to different eGFR equations. eGFR values of all individuals (*n* = 596) based on the different equations assessed in the study. The values are demonstrated as median (with IQR) in boxplots.

Figure 2 shows the median eGFR of each assessed equation as boxplots. The highest eGFR values were obtained using the eGFRcr CKD-EPI 2009 equation with a median eGFR of 54 ml/min/1.73 m^2^ (41–68). In contrast, the eGFRcys CKD-EPI 2012 equation yielded the lowest eGFR values, with a median eGFR of 45 ml/min/1.73 m^2^ (32–59) in the study cohort. Consequently, median eGFR differed up to 9 ml/min/1.73 m^2^. Median eGFR in equations de-indexed for BSA (Appendix 1) were slightly higher.

### Median relative differences between eGFRcr and eGFRcys equations

**Figure 3:**
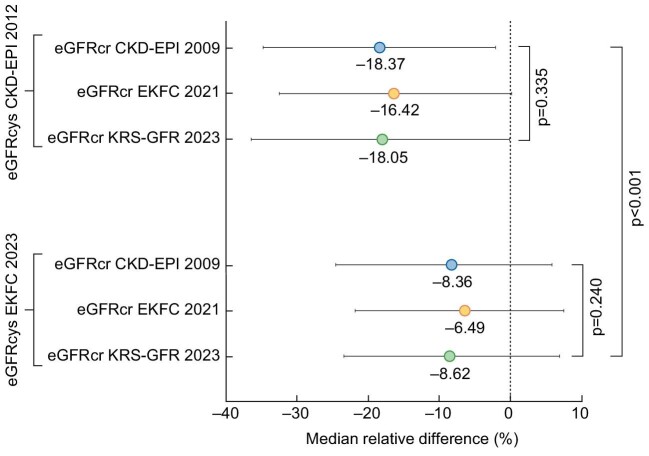
Median relative differences of all eGFRcr equations compared to the references. Visualization of the median relative differences (with IQR) in all individuals (*n* = 596) comparing each eGFRcr equation with the two reference equations (eGFRcys CKD-EPI 2012 and EKFC 2023) as forest plots. For all values, please see Appendix 3.

The median relative differences between all eGFRcr equations compared to the reference equations (eGFRcys) are visualized in Fig. 3 and all median differences (absolute and relative) are listed in Appendix 3. All median relative differences between eGFRcr and eGFRcys equations were negative. Generally, median relative differences were more negative, when comparing several eGFRcr equations to eGFRcys CKD-EPI 2012, than comparing them to eGFRcys EKFC 2023 (Fig. 3, *P* < .001). Within the three analyzed eGFRcr equations EKFC 2021 yielded the closest median values compared to the reference equations (−16.42% compared to eGFRcys CKD-EPI 2012 and −6.49% compared to eGFRcys EKFC 2023), however, not statistically significant when directly compared to the other eGFRcr equations investigated (*P* = .335 and 0.240, respectively).

### Impact of average eGFR on relative differences

**Figure 4:**
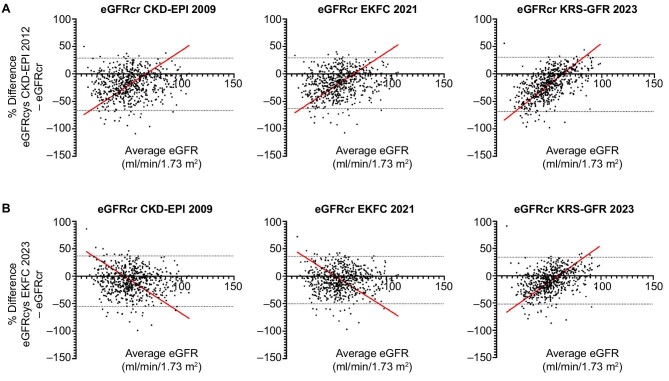
Bland–Altman plots of all eGFRcr equations compared to the references. Relative differences of all individuals (*n* = 596) plotted against the average eGFR between the examined equations. The red line demonstrates a Deming regression of the relative differences, according to different eGFR stages after standardization. The dotted lines visualize the 95% limits of agreement according to the Bland–Altman method. (**A**) Comparisons between eGFRcr equations and eGFRcys CKD-EPI 2012. (**B**) Comparisons between eGFRcr equations and eGFRcys EKFC 2023. For the linear functions of all Deming regressions, please see Appendix 4.

Bland–Altman plots display the relation between relative differences and average eGFR-stages, as shown in Fig. 4. Comparing eGFRcr equations to eGFRcys CKD-EPI 2012 (Fig. 4a), Deming regression lines (as linear functions, demonstrated in red) show a small mean relative difference around an average eGFR of 60 ml/min/1.73 m^2^, and a positive relative difference (eGFRcys > eGFRcr) at higher average eGFR-stages. This applies also for the comparison of eGFRcr KRS-GFR 2023 and eGFRcys EKFC 2023 (Fig. 4b). However, when comparing eGFRcr CKD-EPI 2009 with eGFRcys EKFC (Fig. 4b), or eGFRcr EKFC 2021 with eGFRcys EKFC (Fig. 4b), the Deming regression lines have a negative slope, suggesting a greater (and negative) mean relative difference with higher average eGFR, and therefore less favorable estimation of GFR according to cystatin C (also see Appendix 4).

### Characteristics associated with smaller or greater differences between eGFRcr and eGFRcys equations

**Figure 5:**
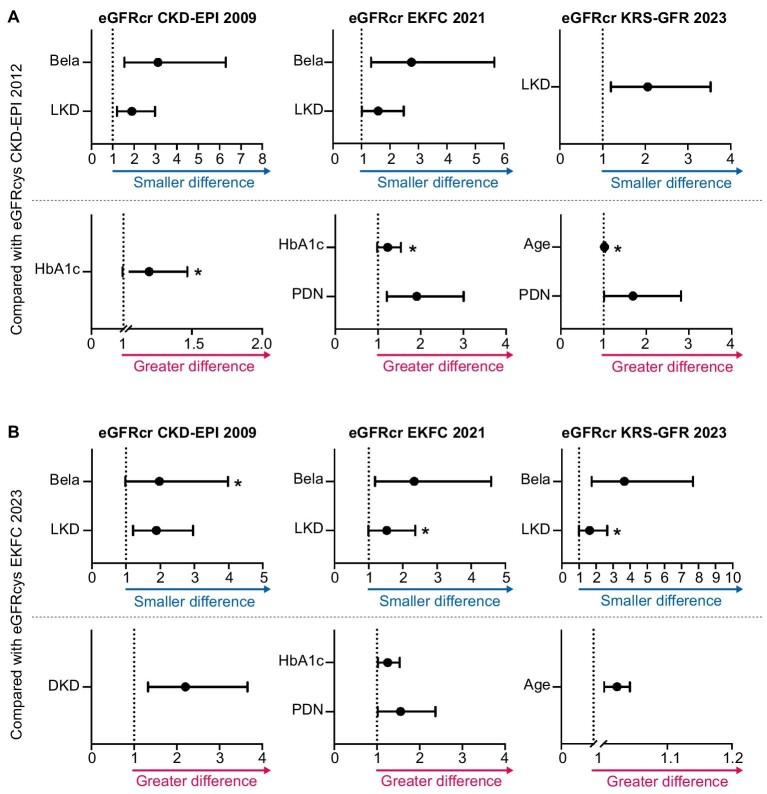
Associations of interest for smaller or greater differences between eGFRcr equations and references. Associations are demonstrated as forest plots with odds ratios (OR) and 95% CI (low and high). (**A**) Comparisons between eGFRcr equations and eGFRcys CKD-EPI 2012. (**B**) Comparisons between eGFRcr equations and eGFRcys EKFC 2023. *P between .05 and .1 (trend, but non-significant). For DKD the reciprocal values (OR, 95% CI) for LKD were calculated. For the exact values, please refer to Table 3. Abbreviations: Bela, belatacept; LKD, living kidney donation; PDN, prednisone; Age, age of the recipient; DKD, deceased kidney donation.

Figure 5 highlights the most relevant transplantation-related characteristics associated with either smaller (≥Q3) or greater (≤Q1) differences between the eGFRcr equations to the reference (eGFRcys). The complete binary logistic regression model can be seen in Table 3. Belatacept use and living kidney donation are independently associated with a smaller difference between eGFRcr and eGFRcys in several scenarios, with OR ranging from 2.332 to 3.661 (belatacept) and 1.585 to 2.056 (living kidney donation). On the contrary, prednisone use has shown to be independently associated with a greater difference between the compared equations in some of the comparisons (OR between 1.554 and 1.907). The median relative differences of subgroups with and without these factors have been independently investigated with Mann–Whitney *U*-test for each comparing scenario (Appendix 5). Other factors are associated with either smaller or greater differences between eGFRcr and eGFRcys, as demonstrated in Table 3: Impaired glycemic control (demonstrated with higher HbA1c-values) is independently associated with a greater difference, comparing eGFRcr EKFC 2021 to eGFRcys EKFC 2023 (OR 1.252, 95% CI 1.023–1.531, *P* = .029) and indicated potential associations in other comparisons, although these did not reach statistical significance (Fig. 5). Interestingly, donor-recipient-sex-mismatch was not independently associated to significant differences between the eGFR equations (only reaching significance in univariable models).

**Table 3:** Binary logistic regression model to assess differences between eGFRcr equations and references.

		Smaller difference with eGFRcys CKD-EPI 2012						Greater difference with eGFRcys CKD-EPI 2012						Smaller difference with eGFRcys EKFC 2023						Greater difference with eGFRcys EKFC 2023					
		Univariable			Multivariable			Univariable			Multivariable			Univariable			Multivariable			Univariable			Multivariable		
Variables	*n* (total)	OR	95% CI	*P* value	OR	95% CI	*P* value	OR	95% CI	*P* value	OR	95% CI	*P* value	OR	95% CI	*P* value	OR	95% CI	*P* value	OR	95% CI	*P* value	OR	95% CI	*P* value
**(a) eGFRcr CKD-EPI 2009**																									
Recipient																									
Sex (male)	359	0.603	(0.415–0.877)	.008	0.643	(0.376–1.099)	.106	1.174	(0.801–1.721)	.412				0.450	(0.309–0.656)	<.001	0.632	(0.351–1.140)	.127	2.057	(1.372–3.085)	<.001	1.404	(0.786–2.507)	.252
Age (years)	-	0.980	(0.966–0.994)	.005	0.989	(0.973–1.006)	.217	1.025	(1.010–1.041)	.001	1.013	(0.996–1.030)	.134	0.977	(0.963–0.991)	.001	0.977	(0.960–0.993)	.007	1.022	(1.007–1.038)	.004	1.014	(0.997–1.030)	.107
BMI (kg/m^2^)	–	0.976	(0.939–1.014)	.218				0.991	(0.954–1.029)	.631				0.972	(0.935–1.010)	.149				1.011	(0.974–1.049)	.560			
BSA (m^2^)	–	0.547	(0.237–1.265)	.159				0.546	(0.236–1.262)	.157				0.480	(0.207–1.113)	.087				1.106	(0.483–2.533)	.811			
HbA1c (%)	–	0.703	(0.544–0.908)	.007	0.841	(0.661–1.070)	.158	1.295	(1.065–1.574)	.009	1.197	(0.976–1.468)	.084	0.661	(0.508–0.861)	.002	0.789	(0.605–1.029)	.080	1.257	(1.035–1.525)	.021	1.132	(0.917–1.398)	.249
Donor																									
Sex (male)	316	1.548	(1.061–2.259)	.023	1.330	(0.714–2.480)	.369	0.750	(0.518–1.088)	.130				1.386	(0.952–2.017)	.089				0.806	(0.556–1.169)	.256			
Age (years)	–	0.995	(0.982–1.007)	.409				1.004	(0.991–1.017)	.530				1.013	(1.000–1.027)	.054				0.998	(0.986–1.011)	.784			
BMI (kg/m^2^)	–	0.951	(0.908–0.997)	.036	0.937	(0.889–0.989)	.018	1.025	(0.982–1.070)	.263				0.983	(0.940–1.028)	.455				0.998	(0.955–1.043)	.918			
Immunosuppression																									
Tacrolimus (yes)	493	0.871	(0.539–1.408)	.574				0.821	(0.510–1.322)	.417				0.653	(0.411–1.037)	.071				1.369	(0.814–2.303)	.236			
Ciclosporin (yes)	45	0.531	(0.232–1.215)	.134				1.556	(0.813–2.979)	.182				0.968	(0.478–1.963)	.929				0.847	(0.409–1.755)	.655			
mTORi (yes)	30	0.586	(0.220–1.560)	.285				1.796	(0.834–3.866)	.135				0.740	(0.296–1.845)	.518				1.796	(0.834–3.866)	.135			
MMF/MPA (yes)	535	1.790	(0.885–3.622)	.105				0.710	(0.399–1.263)	.244				1.406	(0.726–2.721)	.312				1.025	(0.554–1.895)	.938			
Azathioprine (yes)	36	0.358	(0.124–1.029)	.057				1.165	(0.548–2.476)	.692				0.583	(0.238–1.430)	.239				0.583	(0.238–1.430)	.239			
Prednisone (yes)	366	0.564	(0.387–0.820)	.003	0.493	(0.323–0.753)	.001	1.445	(0.976–2.139)	.066				0.910	(0.623–1.330)	.627				1.335	(0.905–1.970)	.146			
Belatacept (yes)	45	2.367	(1.269–4.414)	.007	3.118	(1.547–6.285)	.001	0.734	(0.345–1.562)	.422				2.892	(1.558–5.365)	<.001	1.975	(0.979–3.981)	.057	0.531	(0.232–1.215)	.134			
TPL characteristics																									
LKD (yes)	172	1.938	(1.310–2.866)	<.001	1.894	(1.202–2.985)	.006	0.541	(0.346–0.847)	.007	0.652	(0.407–1.042)	.074	2.183	(1.478–3.226)	<.001	1.888	(1.204–2.960)	.006	0.436	(0.273–0.695)	<.001	0.455**	(0.274–0.753)	.002
DGF (yes)	123	0.809	(0.504.1.300)	.381				0.763	(0.472–1.232)	.268				1.014	(0.642–1.601)	.953				0.718	(0.442–1.166)	.180			
Re-TPL (yes)	83	0.415	(0.214–0.806)	.009	0.501	(0.248–1.011)	.054	1.545	(0.935–2.553)	.089				0.624	(0.345–1.129)	.119				1.545	(0.935–2.553)	.089			
Lab to TPL (days)	–	1.000	(1.000–1.000)	.680				1.000	(1.000–1.000)	.779				1.000	(1.000–1.000)	.902				1.000	(1.000–1.000)	.754			
FF (yes)	109	1.046	(0.649–1.684)	.854				1.243	(0.781–1.978)	.359				1.389	(0.878–2.197)	.161				0.717	(0.431–1.193)	.200			
FM (yes)	171	0.547	(0.350–0.856)	.008	0.772	(0.335–1.777)	.543	1.200	(0.802–1.796)	.374				0.492	(0.312–0.777)	.002	0.620	(0.342–1.127)	.117	1.603	(1.080–2.380)	.019	1.350	(0.835–2.184)	.221
MM (yes)	188	0.959	(0.643–1.432)	.839				1.000	(0.671–1.490)	1.000				0.741	(0.491–1.120)	.155				1.328	(0.898–1.962)	.155			
MF (yes)	128	1.898	(1.243–2.897)	.003	*	*	*	0.633	(0.388–1.032)	.067				2.180	(1.432–3.319)	<.001	1.379	(0.757–2.513)	.294	0.453	(0.267–0.768)	.003	0.666	(0.335–1.325)	.247
D-R-BMI-MM (%)	–	0.998	(0.991–1.006)	.633				1.004	(0.997–1.012)	.267				1.003	(0.995–1.010)	.477				0.998	(0.991–1.006)	.680			
Functional characteristics																									
Proteinuria (mg/day)	–	1.000	(0.999–1.000)	.823				1.000	(1.000–1.001)	.095				1.000	(1.000–1.001)	.553				1.000	(1.000–1.001)	.653			
Average eGFR (ml/min/1.73m2)	–	1.010	(1.000–1.020)	.041	1.004	(0.993–1.015)	.453	0.981	(0.971–0.991)	<.001	0.984	(0.974–0.995)	.005	0.986	(0.976–0.996)	0.006	0.979	(0.968–0.991)	<.001	1.006	(0.996–1.016)	.255			
D Lowest SCr (µmol/l)	–	1.766	(1.020–3.056)	.042	1.615	(0.827–3.155)	.161	0.877	(0.484–1.589)	.665				1.763	(1.018–3.051)	0.043	1.514	(0.803–2.853)	.200	0.653	(0.347–1.229)	.186			
**(b) eGFRcr EKFC 2021**																									
Recipient																									
Sex (male)	359	0.582	(0.400–0.846)	.005	0.521	(0.276–0.982)	.044	1.317	(0.895–1.937)	.162				0.347	(0.237–0.507)	<0.001	0.301	(0.165–0.550)	<.001	2.243	(1.488–3.380)	<.001	1.826	(1.001–3.331)	.050
Age (years)	–	0.994	(0.980–1.009)	.431				1.013	(0.998–1.028)	.082				0.988	(0.974–1.002)	.086				1.010	(0.995–1.024)	.199			
BMI (kg/m^2^)	–	0.959	(0.922–0.998)	.039	0.936	(0.882–0.993)	.030	0.978	(0.941–1.016)	.248				0.979	(0.942–1.017)	.268				1.015	(0.978–1.053)	.442			
BSA (m^2^)	–	0.429	(0.184–0.998)	.049	2.155	(0.479–9.706)	.317	0.737	(0.320–1.694)	.472				0.336	(0.143–0.788)	.012	1.382	(0.478–3.995)	.551	1.323	(0.578–3.027)	.507			
HbA1c (%)	–	0.771	(0.604–0.983)	.036	0.882	(0.700–1.111)	.287	1.272	(1.047–1.545)	.015	1.228	(0.981–1.536)	.073	0.691	(0.533–0.895)	.005	0.759	(0.583–0.987)	.040	1.309	(1.077–1.592)	.007	1.252	(1.023–1.531)	.029
Donor																									
Sex (male)	316	1.668	(1.141–2.439)	.008	1.466	(0.795–2.701)	.220	0.698	(0.481–1.013)	.059				1.198	(0.825–1.739)	.344				0.836	(0.577–1.211)	.344			
Age (years)	–	0.990	(0.978–1.003)	.134				1.006	(0.993–1.019)	.358				1.007	(0.994–1.020)	.300				0.994	(0.981–1.006)	.320			
BMI (kg/m^2^)	–	0.966	(0.923–1.011)	.138				1.030	(0.987–1.076)	.179				0.976	(0.933–1.021)	.290				0.996	(0.953–1.041)	.859			
Immunosuppression																									
Tacrolimus (yes)	493	0.926	(0.570–1.503)	.755				0.871	(0.539–1.408)	.574				0.774	(0.483–1.242)	.288				1.471	(0.867–2.493)	.152			
Ciclosporin (yes)	45	0.629	(0.286–1.382)	.248				1.240	(0.632–2.430)	.532				0.629	(0.286–1.382)	.248				0.847	(0.409–1.755)	.655			
mTORi (yes)	30	0.586	(0.220–1.560)	.285				1.796	(0.834–3.866)	.135				0.740	(0.296–1.845)	.518				1.796	(0.834–3.866)	.135			
MMF/MPA (yes)	535	1.406	(0.726–2.721)	.312				0.599	(0.341–1.053)	.075				1.580	(0.800–3.121)	.188				0.775	(0.432–1.390)	.392			
Azathioprine (yes)	36	0.583	(0.238–1.430)	.239				1.346	(0.645–2.805)	.429				0.583	(0.238–1.430)	.239				0.849	(0.378–1.906)	.692			
Prednisone (yes)	366	0.524	(0.360–0.762)	<.001	0.469	(0.308–0.714)	<.001	1.701	(1.141–2.535)	.009	1.907	(1.209–3.006)	.005	0.785	(0.538–1.144)	.207				1.567	(1.055–2.326)	.026	1.554	(1.019–2.371)	.041
Belatacept (yes)	45	1.927	(1.023–3.632)	.042	2.755	(1.339–5.668)	.006	0.968	(0.478–1.963)	.929				2.617	(1.408–4.866)	.002	2.332	(1.186–4.587)	.014	0.531	(0.232–1.215)	.134			
TPL characteristics																									
LKD (yes)	172	1.650	(1.113–2.447)	.013	1.585	(1.012–2.483)	.044	0.729	(0.476–1.115)	.145				1.718	(1.160–2.546)	.007	1.530	(0.990–2.365)	.056	0.487	(0.308–0.769)	.002	0.514	(0.316–0.835)	.007
DGF (yes)	123	0.809	(0.504–1.300)	.381				0.633	(0.385–1.041)	.072				1.014	(0.642–1.601)	.953				0.718	(0.442–1.166)	.180			
Re-TPL (yes)	83	0.324	(0.158–0.665)	.002	0.368	(0.172–0.786)	.010	1.545	(0.935–2.553)	.089				0.624	(0.345–1.129)	.119				1.759	(1.072–2.886)	.025	1.558	(0.913–2.661)	.104
Lab to TPL (days)	–	1.000	(1.000–1.000)	.959				1.000	(1.000–1.000)	.685				1.000	(1.000–1.000)	.723				1.000	(1.000–1.000)	.774			
FF (yes)	109	1.046	(0.649–1.684)	.854				1.109	(0.691–1.778)	.669				1.721	(1.098–2.696)	.018	0.780	(0.449–1.355)	.377	0.669	(0.399–1.122)	.128			
FM (yes)	171	0.492	(0.312–0.777)	.002	0.735	(0.317–1.704)	.473	1.418	(0.953–2.111)	.085				0.492	(0.312–0.777)	.002	0.857	(0.475–1.544)	.607	1.603	(1.080–2.380)	.019	1.206	(0.747–1.949)	.444
MM (yes)	188	1.000	(0.671–1.490)	1.000				0.959	(0.643–1.432)	.839				0.536	(0.348–0.826)	.005	*	*	*	1.437	(0.974–2.120)	.068			
MF (yes)	128	1.988	(1.303–3.032)	.001	*	*	*	0.595	(0.362–0.976)	.040	0.583	(0.321–1.059)	.077	2.501	(1.646–3.799)	<.001	*	*	*	0.421	(0.246–0.721)	.002	0.749	(0.366–1.532)	.428
D-R-BMI-MM (%)	–	1.002	(0.994–1.010)	.590				1.007	(0.999–1.015)	.088				1.001	(0.993–1.008)	.851				0.998	(0.990–1.005)	.575			
Functional characteristics																									
Proteinuria (mg/day)	–	1.000	(0.999–1.000)	.165				1.000	(1.000–1.001)	.019	1.000	(1.000–1.001)	.265	1.000	(1.000–1.001)	.631				1.000	(1.000–1.001)	.302			
Average eGFR (ml/min/1.73 m^2^)	–	1.023	(1.012–1.033)	<.001	1.024	(1.012–1.035)	<.001	0.969	(0.958–0.980)	<.001	0.972	(0.960–0.985)	<.001	0.997	(0.987–1.007)	.566				1.000	(0.990–1.010)	.995			
D Lowest SCr (µmol/l)	–	1.704	(0.985–2.950)	.057				0.990	(0.554–1.770)	.973				1.537	(0.886–2.666)	.126				0.650	(0.345–1.224)	.182			
**(c) eGFRcr KRS-GFR 2023**																									
Recipient																									
Sex (male)	359	0.839	(0.576–1.221)	.359				0.903	(0.619–1.316)	.595				0.503	(0.345–0.731)	<.001	0.624	(0.333–1.170)	.142	1.424	(0.966–2.100)	.075			
Age (years)	–	0.969	(0.955–0.983)	<.001	0.995	(0.976–1.015)	.639	1.036	(1.020–1.052)	<.001	1.021	(0.999–1.043)	.062	0.964	(0.950–0.978)	<.001	0.977	(0.959–0.995)	.012	1.041	(1.025–1.058)	<.001	1.022	(1.002–1.042)	.032
BMI (kg/m^2^)	–	0.973	(0.936–1.012)	.172				0.995	(0.958–1.033)	.775				0.964	(0.926–1.002)	.065				0.994	(0.957–1.032)	.759			
BSA (m^2^)	–	0.635	(0.276–1.465)	.287				0.629	(0.273–1.451)	.277				0.377	(0.162–0.881)	.024	1.099	(0.354–3.410)	.870	0.737	(0.321–1.695)	.473			
HbA1c (%)	–	0.834	(0.662–1.051)	.125				1.342	(1.103–1.633)	.003	1.215	(0.935–1.578)	.146	0.790	(0.621–1.003)	.053				1.243	(1.024–1.509)	.028	1.046	(0.817–1.338)	.723
Donor																									
Sex (male)	316	1.492	(1.023–2.175)	.038	1.539	(0.946–2.503)	.082	0.724	(0.499–1.050)	.089				1.492	(1.023–2.175)	.038	1.129	(0.601–2.123)	.706	0.866	(0.598–1.256)	.449			
Age (years)	–	0.966	(0.954–0.979)	<.001	1.000	(0.980–1.020)	1.000	1.021	(1.007–1.035)	.003	0.985	(0.965–1.005)	.151	0.976	(0.964–0.989)	<.001	1.015	(0.997–1.033)	.110	1.019	(1.006–1.033)	.005	0.991	(0.973–1.009)	.314
BMI (kg/m^2^)	–	0.931	(0.887–0.976)	.003	0.964	(0.906–1.027)	.258	1.044	(1.000–1.090)	.048	1.039	(0.982–1.099)	.186	0.929	(0.885–0.975)	.003	0.929	(0.877–0.984)	.012	1.034	(0.990–1.079)	.135			
Immunosuppression																									
Tacrolimus (yes)	493	1.118	(0.678–1.843)	.662				0.653	(0.411–1.037)	.071				0.985	(0.604–1.606)	.950				0.821	(0.510–1.322)	.417			
Ciclosporin (yes)	45	0.734	(0.345–1.562)	.422				1.927	(1.023–3.632)	.042	1.589	(0.682–3.702)	.283	0.531	(0.232–1.215)	.134				1.734	(0.914–3.291)	.092			
mTORi (yes)	30	0.909	(0.382–2.163)	.829				1.796	(0.834–3.866)	.135				0.586	(0.220–1.560)	.285				1.536	(0.702–3.360)	.283			
MMF/MPA (yes)	535	1.580	(0.800–3.121)	.188				0.651	(0.368–1.152)	.141				1.406	(0.726–2.721)	.312				0.651	(0.368–1.152)	.141			
Azathioprine (yes)	36	0.358	(0.124–1.029)	.057				1.165	(0.548–2.476)	.692				0.583	(0.238–1.430)	.239				1.165	(0.548–2.476)	.692			
Prednisone (yes)	366	0.677	(0.465–0.986)	.042	0.772	(0.476–1.251)	.293	1.849	(1.236–2.768)	.003	1.687	(1.010–2.815)	.046	0.629	(0.432–0.916)	.016	0.527	(0.336–0.828)	.005	1.389	(0.940–2.052)	.099			
Belatacept (yes)	45	1.240	(0.632–2.430)	.532				0.847	(0.409–1.755)	.655				1.927	(1.023–3.632)	.042	3.661	(1.746–7.677)	<.001	0.629	(0.286–1.382)	.248			
TPL characteristics																									
LKD (yes)	172	1.718	(1.160–2.546)	.007	2.056	(1.197–3.532)	.009	0.600	(0.387–0.930)	.023	0.768	(0.435–1.354)	.361	1.650	(1.113–2.447)	.013	1.625	(0.995–2.655)	.052	0.487	(0.308–0.769)	.002	0.588	(0.341–1.012)	.055
DGF (yes)	123	0.857	(0.536–1.371)	.521				0.809	(0.504–1.300)	.381				0.960	(0.605–1.522)	.861				0.675	(0.413–1.102)	.116			
Re-TPL (yes)	83	0.415	(0.214–0.806)	.009	0.480	(0.211–1.093)	.081	1.874	(1.145–3.067)	.012	1.694	(0.899–3.192)	.103	0.464	(0.244–0.882)	.019	0.477	(0.232–0.982)	.044	1.352	(0.811–2.253)	.247			
Lab to TPL (days)	–	1.000	(1.000–1.000)	.840				1.000	(1.000–1.000)	.326				1.000	(1.000–1.000)	.646				1.000	(1.000–1.000)	.182			
FF (yes)	109	0.871	(0.533–1.423)	.582				1.389	(0.878–2.197)	.161				1.466	(0.929–2.314)	.100				0.818	(0.498–1.344)	.427			
FM (yes)	171	0.669	(0.434–1.031)	.068				1.150	(0.768–1.724)	.497				0.416	(0.259–0.667)	<.001	0.485	(0.201–1.168)	.107	1.361	(0.913–2.028)	.130			
MM (yes)	188	1.178	(0.794–1.746)	.416				0.775	(0.514–1.168)	.223				0.959	(0.643–1.432)	.839				1.086	(0.731–1.614)	.684			
MF (yes)	128	1.426	(0.925–2.199)	.108				0.849	(0.535–1.350)	.490				1.811	(1.184–2.769)	.006	*	*	*	0.715	(0.444–1.152)	.168			
D-R-BMI-MM (%)	–	0.997	(0.990–1.004)	.423				1.006	(0.998–1.014)	.154				0.998	(0.991–1.006)	.608				1.004	(0.996–1.012)	.334			
Functional characteristics																									
Proteinuria (mg/day)	–	0.999	(0.999–1.000)	.036	1.000	(0.999–1.001)	.919	1.001	(1.000–1.001)	<.001	1.000	(1.000–1.001)	.353	1.000	(0.999–1.000)	.282				1.001	(1.000–1.001)	.008	1.000	(1.000–1.001)	.483
Average eGFR (ml/min/1.73 m^2^)	–	1.077	(1.061–1.093)	<.001	1.074	(1.054–1.095)	<.001	0.925	(0.910–0.940)	<.001	0.932	(0.913–0.950)	<.001	1.054	(1.041–1.069)	<.001	1.058	(1.040–1.076)	<.001	0.949	(0.936–0.963)	<.001	0.953	(0.937–0.970)	<.001
D lowest SCr (µmol/l)	–	1.589	(0.917–2.753)	.099				1.112	(0.629–1.965)	.716				1.466	(0.844–2.546)	.174				0.957	(0.533–1.717)	.882			

Abbreviations: mTORi, mTOR inhibitors; MMF/MPA, mycophenolate; LKD, living kidney donation; DGF, delayed graft function; Re-TPL, repeat transplantation; FF, female to female donation; FM, female to male donation; MM, male to male donation; MF, male to female donation; D-R-BMI-MM, donor-recipient-BMI-mismatch; D Lowest SCr, lowest serum creatinine of the donor.

All variables have been tested in univariable models and have been included in the multivariable model, when *P* was <.05. To avoid multicollinearity, variables with correlation *r* > .7 have not been analyzed together.*Excluded due to redundancy.**Reciprocal values of OR and 95% CI for deceased kidney donation (DKD) are 2.198 (1.328–3.650).

## DISCUSSION

Using creatinine and cystatin C for GFR estimation in KTRs potentially improves the assessment of ‘real’ kidney function. However, this approach has been extensively debated in historical and recent studies [25–27, 28]. While measuring GFR using Iohexol plasma clearance or scintigraphic tools such as technetium-labelled diethylene-triamine-pentacetate (99mTc-DTPA) is an accurate alternative to eGFR, it is time-consuming and not feasible for routine clinical practice. By contrast, evaluating cystatin C, much like creatinine, involves a simple blood draw. However, cystatin C is relevantly more expensive than creatinine and not universally available, yet it could improve the clinical management in uncertain situations [29]. Here, we evaluated the discrepancies between eGFRcr and eGFRcys in the setting of kidney transplantation, incorporating the most recent, specifically tailored equation eGFRcr KRS-GFR 2023 into the analysis.

Overall, median eGFR varied up to 9 ml/min/1.73 m^2^ throughout the investigated equations. This is important, since many therapeutic decisions rely on eGFR. De-indexing eGFR and calculating it with the consideration of BSA revealed slightly better median GFR. It seems, from other studies, that de-indexing is particularly important in terms of correct drug dosing (e.g. in obese patients) [30]. As expected, also in our kidney transplantation cohort, median eGFRcys was generally lower than median eGFRcr, in line with findings from previous studies that examined CKD patients [31]. Median relative differences between eGFRcr and eGFRcys were similar when comparing the three different eGFR equations based on creatinine. These findings contribute to the ongoing discussion about the necessity of the new KRS-GFR 2023; considering its development may appear redundant, particularly since it does not include cystatin C and its validation relies solely on mGFR [32].

Relative differences between eGFRcr and eGFRcys were influenced by eGFR (here, average eGFR) itself, as demonstrated by Bland–Altman plots. While this discovery is not groundbreaking, our study underscores the significance of this impact within the kidney transplant community. The greatest change of the mean relative difference according to different eGFR-stages was observed between eGFRcr KRS-GFR 2023 and eGFRcys CKD-EPI 2012, showing a steep slope of 1.515 in the linear function (Deming regression model; Appendix 4). Interestingly, when comparing eGFRcr CKD-EPI 2009 with eGFRcys EKFC 2023, the Deming regression demonstrates greater relative differences at higher average eGFR-stages, with a less favorable estimation of GFR based on cystatin C at higher eGFR. This possibly reflects the fact that the eGFRcys EKFC 2023 equation does not account for sex.

In our study, we discovered that specific immunosuppressive medications independently affect the differences between eGFRcr and eGFRcys equations. Patients receiving belatacept as their main immunosuppression (avoiding calcineurin inhibitors) showed a significant association with smaller differences between several eGFRcr equations compared to eGFRcys equations. The effect is independent of eGFR-stages. It remains unclear whether this effect stems directly from the drug or is due to the absence of calcineurin inhibitor-effects (on creatinine). It also remains to be investigated whether the effect of an improvement in renal function under belatacept is ultimately solely a creatinine effect, and according to cystatin C: for example, no improvement in eGFR can be objectively observed. The other group not using calcineurin inhibitors as their primary immunosuppressants (mammalian target of rapamycin inhibitors) was relatively small and mixed, limiting further conclusive observations. Conversely, the use of low dose prednisone as maintenance immunosuppression was associated with greater differences between eGFRcr and eGFRcys equations. The influence of prednisone on creatinine can be attributed to catabolic effects (muscle loss), which may lead to falsely low creatinine levels resulting in an overestimation of GFR [33]. In contrast to creatinine, cystatin C production appears to be increased in various tissues with corticosteroid treatment, resulting in an underestimation of GFR. This phenomenon has been observed in animal models as well as in KTRs and is likely mediated by the corticosteroid's effect on gene transcription [34], [35]. These observations taken together are explaining the greater divergence between eGFRcr and eGFRcys in individuals with corticosteroids. In this study, unfortunately, we only considered body mass index (BMI) and body surface area (BSA) as potential indicators for body composition, whereas other measurements assessing muscle status, such as bioelectrical impedance analysis or radiological studies such as computed tomography or magnetic resonance imaging were not available. In our analysis, lower BMI was only independently associated with a smaller difference between eGFRcr and eGFRcys, when comparing eGFRcr EKFC 2021 with eGFRcys CKD-EPI 2012. Further research is needed on how muscle mass directly affects differences between eGFRcr and eGFRcys. High HbA1c (reflecting glycemic control) and repeat transplantation showed a trend toward impacting the difference between eGFRcr and eGFRcys in the logistic regression model. Repeat transplantation has been associated with a greater difference between creatinine and cystatin C levels, as well as a higher risk of death with functioning graft. [36] This trend is likely because these patients potentially mismanaged their previous graft or received a suboptimal kidney in the earlier transplantation. Last, we observed that living kidney donation independently predicted a narrower variance in most comparisons. This outcome may be attributed to confounding factors: generally, living kidney donation is associated with better organ quality reflected by higher nephron mass, shorter ischemia times, and lower inflammation [37, 38, 39]. Additionally, there appears to be a selection bias among KTRs, as those receiving living kidney transplants (especially in a pre-emptive setting) are more often individuals of higher socioeconomic status (SES), and generally have fewer metabolic comorbidities and higher muscle mass [40]. In the general population, higher SES has been associated with better kidney function and a reduced prevalence of metabolic disorders, which also contribute to the variation between eGFR equations [41].

Despite the detailed presentation of transplantation-related characteristics, certain limitations of our study persist. The application of the Jaffe method for assessment of creatinine holds the potential problem of interference with other biomarkers or drugs and can differ significantly from other established measuring methods, such as the enzymatic method [42]. However, throughout the study, the method (Jaffe) has never been changed to another method. Furthermore, this investigation did not include mGFR or creatinine clearance through 24-hour urine collections. Nevertheless, using cystatin C as an additional validation tool holds practicality for everyday practice compared to mGFR. As most of the studied population were White, statements about race coefficients could not be addressed. Yet, the displayed impact of average eGFR-stages themselves, or transplantation-related variables, such as belatacept use, prednisone use, or living kidney donation as independent factors influencing the difference between eGFRcr and eGFRcys equations is new.

## CONCLUSIONS

Our investigation revealed that the discrepancy between eGFRcr and eGFRcys is influenced by eGFR itself, along with certain transplantation-related characteristics and immunosuppressive medications. Our study highlights clinical scenarios where the assessment of kidney function using creatinine may differ significantly from that using cystatin C, such as with prednisone use, indicating the need for additional validation tools to ensure accurate classification into CKD stages. Conversely, in cases such as living kidney donation or the use of belatacept, eGFRcr, and eGFRcys tend to correlate more closely. Follow-up studies are essential to confirm the findings demonstrated in our analysis.

## Supplementary Material

sfae253_Supplemental_File

## Data Availability

The data that support the findings of this study are available upon request from the corresponding author, T.S. The data are not publicly available due to restrictions e.g. their containing information that could compromise the privacy of research participants.
