# Supplementary material for: Immunosuppression and transplantation-related characteristics affect the difference between eGFR equations based on creatinine compared to cystatin C in kidney transplant recipients
Source: Clin Kidney J. 2024 Aug 20;17(11):sfae253. doi: 10.1093/ckj/sfae253 (PMC11536772; doi:10.1093/ckj/sfae253)

**SUPPLEMENTARY MATERIALS (APPENDIX)**

1. **Appendix 1:** Median de-indexed eGFR values according to different eGFR equations
2. **Appendix 2:** Correlation matrix of all variables tested in the regression model
3. **Appendix 3:** Median differences for all eGFRcr equations compared to the references
4. **Appendix 4:** Deming regression and linear functions of mean average eGFR
5. **Appendix 5:** Median relative differences in different subgroups

|  | **Median de-indexed eGFR (ml/min/BSA)** |
| --- | --- |
| eGFRcr CKD-EPI 2009 | 57 (43-74) |
| eGFRcr EKFC 2021 | 56 (42-71) |
| eGFRcr KRS-GFR 2023 | 56 (46-68) |
| eGFRcys CKD-EPI 2012 | 48 (34-62) |
| eGFRcys EKFC 2023 | 52 (39-66) |

**Appendix 1 |** **Median de-indexed eGFR values according to different eGFR equations:** eGFR values of all individuals (n=596) based on the different equations assessed in the study and de-indexed for body surface area (BSA). The values are demonstrated as median and interquartile ranges.

**Appendix 2 |** **Correlation matrix of all variables tested in the regression model:** Variables with a r-value >0.7 are marked in red (only two correlations). Spearman-Rho test was applied.

|  | **eGFRcys CKD-EPI 2012 (reference)** | |
| --- | --- | --- |
|  | Median abs. difference | Median rel. difference |
| eGFRcr CKD-EPI 2009 | -7.79 (-15.83, -1.11) | -18.37 (-34.71, -2.15) |
| eGFRcr EKFC 2021 | -7.14 (-14.43, 0.04) | -16.42 (-32.36, 0.15) |
| eGFRcr KRS-GFR 2023 | -8.09 (-14.48, -0.08) | -18.05 (-36.30, -0.15) |
|  |  |  |
|  | **eGFRcys EKFC 2023 (reference)** | |
|  | Median abs. difference | Median rel. difference |
| eGFRcr CKD-EPI 2009 | -4.02 (-11.73, 2.69) | -8.36 (-24.41, 5.75) |
| eGFRcr EKFC 2021 | -3.18 (-10.39, 3.28) | -6.49 (-21.77, 7.27) |
| eGFRcr KRS-GFR 2023 | -3.84 (-10.48, 3.95) | -8.62 (-23.39, 6.77) |

**Appendix 3** **| Median differences for all eGFRcr equations compared to the references:** Median absolute differences are calculated as eGFRcys minus eGFRcr. Median relative differences are calculated as eGFRcys minus eGFRcr divided by the average of both and multiplied with 100%. Abbreviations: *abs., absolute; rel., relative*.

|  | **Deming regression function** | **p-value** |
| --- | --- | --- |
| **eGFRcr CKD-EPI 2009 with eGFRcys CKD-EPI 2012** | $y=1.261*x-83.54$ | <0.001 |
| **eGFRcr EKFC 2021 with eGFRcys CKD-EPI 2012** | $y=1.261*x-80.73$ | <0.001 |
| **eGFRcr KRS-GFR 2023 with eGFRcys CKD-EPI 2012** | $y=1.515*x-95.25$ | <0.001 |
| **eGFRcr CKD-EPI 2009 with eGFRcys EKFC 2023** | $y=-1.245*x+56.90$ | 0.018 |
| **eGFRcr EKFC 2021 with eGFRcys EKFC 2023** | $y=-1.210*x+56.09$ | 0.834 |
| **eGFRcr KRS-GFR 2023 with eGFRcys EKFC 2023** | $y=1.354*x-79.91$ | <0.001 |

**Appendix 4 |** **Deming regression and linear functions of mean average eGFR:** Deming regression has been performed in each Bland-Altman plot after standardization of the y- and x-axis (with standard deviation). The deming regression functions are demonstrated as a linear function (y=m*x+q), where m is the slope and q the y-intercept. The p-values indicate if the function is significantly «non-zero».

**Appendix 5 |** **Median relative differences in different subgroups:** The median relative differences were analyzed separately in subgroups categorized by the presence or absence of belatacept (top), prednisone (middle), or living kidney donation (bottom). Each scenario was compared independently within these groups. Mann-Whitney U test was performed. Abbreviations: *LKD, living kidney donation; DKD, deceased kidney donation.*


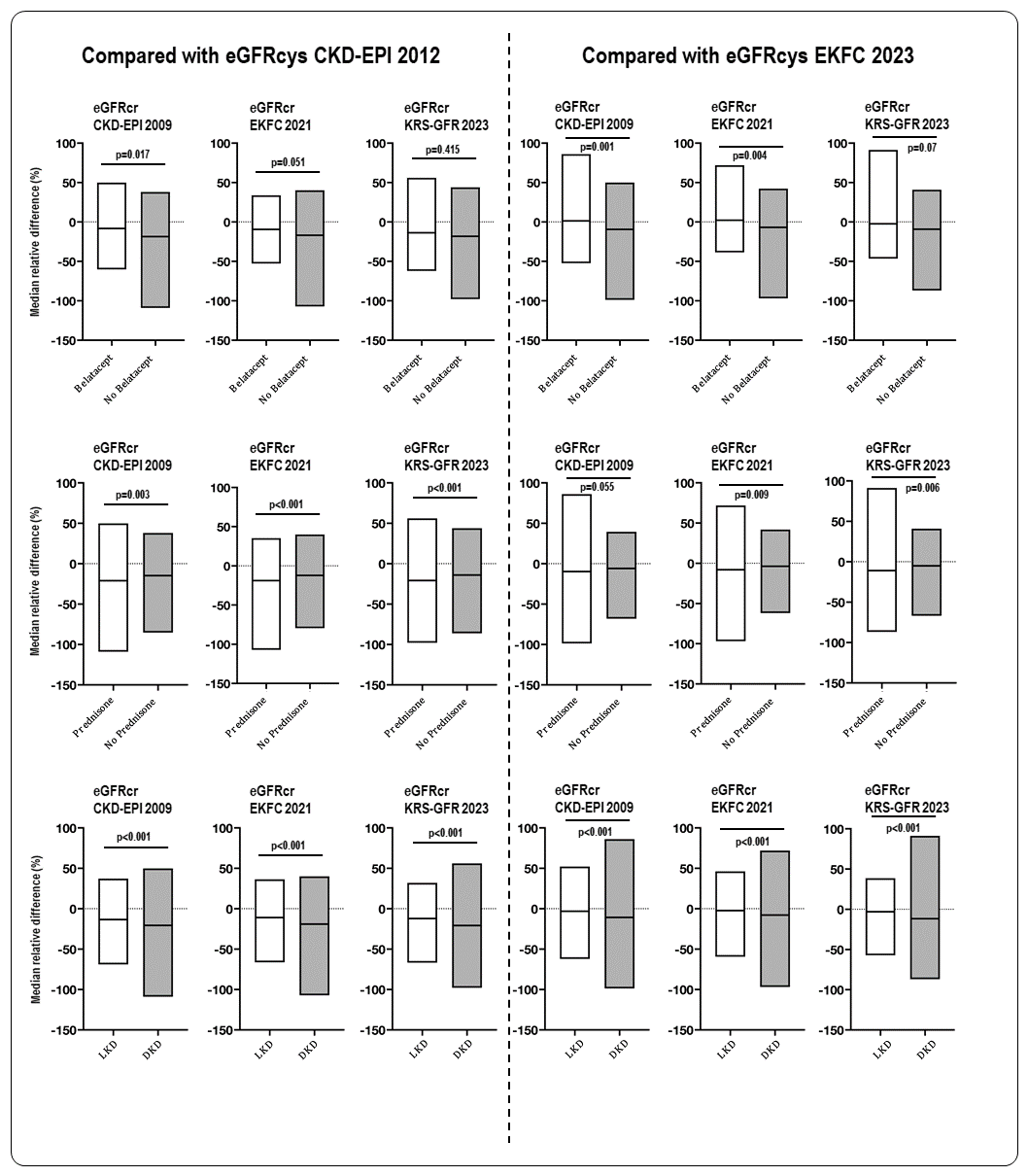

Supplement: sfae253_Supplemental_File [file sfae253_supplemental_file.docx]
